# Assessment of External Load during Matches in Two Consecutive Seasons Using the Mediacoach^®^ Video Analysis System in a Spanish Professional Soccer Team: Implications for Injury Prevention

**DOI:** 10.3390/ijerph18031128

**Published:** 2021-01-27

**Authors:** Manuel Alcantarilla-Pedrosa, David Álvarez-Santana, Sergio Hernández-Sánchez, Angel Yañez-Álvarez, Manuel Albornoz-Cabello

**Affiliations:** 1 Medical Department of Real Betis Balompié S.A.D, Avda. Heliópolis, s/n, 41012 Sevilla, Spain; malcped@ay360grados.com (M.A.-P.); alvsandavid@gmail.com (D.Á.-S.); 2 Translational Research Center of Physiotherapy, Department of Pathology and Surgery, Miguel Hernandez University, 03550 Sant Joan, Alicante, Spain; 3 Department of Physiotherapy, Faculty of Nursing, Physiotherapy, and Podiatry, University of Seville, 41009 Seville, Spain; ayalvarez@us.es (A.Y.-Á.); malbornoz@us.es (M.A.-C.)

**Keywords:** soccer, injury, video-analysis, match-performance, prevention

## Abstract

(1) Background: Knowledge of competition loads is a relevant aspect of injury prevention. We aimed to describe the training and match injury incidence and physical demand variables observed during a competition using a multi-camera video analysis system (Mediacoach^®^) (LaLiga^TM^, Madrid, Spain) in a professional Spanish soccer team during two consecutive seasons. (2) Methods: 30 players (age: 26.07 ± 3.78 years) participated in the study. Physical variables of 74 matches were collected retrospectively. Injury characteristics of both seasons were also collected. Differences in these variables between the two seasons and by player position and correlations between variables were explored. (3) Results: There were statistically significant differences between the two seasons in the total distance traveled and the distance traveled at a high-intensity sprint (*p* < 0.05). During the two seasons, there was an average of 4.7 ± 2.2 injuries. The total distance traveled was different according to the playing position, and statistically significant correlations were found in the total distance and sprint at a high intensity for certain positions with different injury severity (4) Conclusions: The match performance data recorded by the Mediacoach^®^ system may provide relevant information by player position to technical and medical staff for injury prevention.

## 1. Introduction

Soccer injuries have a major impact on the performance of both the player and the team due to the concerned player’s loss of training sessions and matches [1,2,3]. It has been reported that a professional soccer team of 25 players suffers approximately 50 injuries per season, which is equivalent to two injuries per player in each season [4]. Furthermore, soccer injuries directly affect the economy of the soccer clubs, with an average cost per injured player per month of about 500,000 euros [5].

A recent meta-analysis shows that the general incidence in male professional soccer players was 8.1 injuries/1000 h of exposure, not varying significantly between professional teams of different leagues and levels. This incidence was 10 times higher in matches than in training (36 injuries/1000 h in matches versus 3.7 injuries/1000 h in training) with the lower limb being the area that records more injuries (6.8 injuries/1000 h of exposure) and muscular/ligamentary being the most common injuries (4.6 injuries/1000 h of exposure) [6].

Over the past 20 years, international organisations, such as the Fédération Internationale de Football Association (FIFA) and the Union of European Football Associations (UEFA), have shown interest in studying different variables related to soccer injuries, including physical demands [7,8], because in recent years, soccer has experienced an increase in physical demands, especially in high-intensity actions [9]. Therefore, it is important to monitor the match demands to detect the potential risk of injury [10,11,12,13].

There are different methods of measuring the physical demands of the player during matches, such as the use of multi-camera video analysis systems or Global Positioning Systems (GPS). Several competitions worldwide have been using different video analysis devices validated and compared to GPS, such as Mediacoach^®^, Amisco (Sport-Universal Process, Nice, France), or Prozone^®^ (Prozone Ltd., Leeds, UK). All of these devices are based on multiple high-definition cameras that are distributed around the stadium to track players’ position in the soccer field. They provide information on physical variables such as distances covered at different intensities, accelerations or decelerations, and others of a technical nature like ball possession [14,15,16,17]. As a result, performance can be analyzed according to the player’s position within his tactical context in order to create specific strategies or interventions in each of them [18,19,20]. They have been described in different European professional leagues such as La Liga (Spain) [21], Serie A (Italy) [22], or the Premier-League (England) [23,24].

Furthermore, external and internal load monitoring provides useful information for injury prevention as it is a variable that significantly affects the player’s performance and fatigue [25,26,27,28,29]. Besides, other factors, such as the coach’s leadership [30], technical quality [31], playing style [32], results [33], category [34], type of match [35], congestion of the calendar [36], or fatigue [37,38], can potentially influence the risk of injury.

Emerging evidence targets load (external and internal) management programs as an interesting tool to reduce injury risk among soccer players [39]. Moreover, considering the higher injury incidence rates during matches in male professional soccer players, Pfirrmann et al. [40] recommended the review of those data in competition to reduce the overall injury rates. Taking into account the data collected about physical demands during the match development, staff members can program actions or even targeted specific preventive exercises.

Therefore, the present study aimed to describe the training and match injury records and physical demand variables registered during competition with the Mediacoach^®^ system in players of a professional soccer team in Spanish La Liga during two consecutive seasons. Besides, the potential differences in physical demand variables and injury incidence were analyzed between seasons and by player position.

## 2. Materials and Methods

### 2.1. Participants

The study was conducted during two consecutive seasons (2012/2013 and 2013/2014). A total of 30 professional players from Spanish La Liga participated in the study (range: 19–32): eight played the 2012/2013 season, 12 played the 2013/2014 season, and 10 played both seasons. In the first season, they played two competitions, namely, La Liga and the Copa del Rey, while in the second one, they played three competitions, namely, La Liga, the Copa del Rey, and the UEFA Europa League. Only the players who participated in the whole season were included, that is, those who joined the team from the corresponding pre-season. Goalkeepers and players who arrived in the second half of the season or were injured before the study period were excluded.

### 2.2. Procedure

Data from the injury register were obtained in the two seasons, from July to the end of May, and therefore the pre-season and the competitive period were included. They were collected retrospectively from the club’s database and subsequently reordered. All analyzed matches were played on natural grass. The collection of information was performed with the written consent of the entity and under an agreement with the university to which the principal researcher belongs as well as with the written consent of each player following the ethical standards of the Declaration of Helsinki. The study protocol for data collection was approved by the Local Ethical Research Committee (project code DPS. 01/14, approval date: 7 February 2014).

A training session was defined as any physical activity scheduled by the coach with the team. A match was defined as any friendly or competitive match. An injury was defined as one that occurs during a training session or match and prevents the player from performing the next training session or match [39].

The classification of the Consensus Statement for Soccer Injuries by Fuller [28] was performed in terms of the type of injury for a better description and later analysis of the results, categorising seven groups into the four following groups: muscle-tendinous, joint-ligamentary, bony, and others.

Injury severity was defined in terms of the number of days from the time the player was injured until he returned and performed a full training session with the team in optimal condition to compete. Based on other studies as a reference [28,41], injury severity was classified into the four following groups: minor (1–3 days), mild (4–7 days), moderate (8–28 days), and severe (>28 days).

The location of the injury has been collected and categorized into 12 groups: head/face/cervical region, sternum/ribs/dorsal region, shoulder/clavicle, upper limbs, abdomen, lumbar/sacral/pelvis, hip/ingle, thigh, knee, leg, ankle, and foot [28,29].

The exposure time of all training sessions and league matches was collected through the club’s database from July to the end of the competitive season in both seasons. The monthly exposure was calculated for each player and then divided by 60 to obtain the exposure in hours. The incidence was expressed as the number of injuries per 1000 h of training or competition.

The variables of the players’ movement patterns were obtained from La Liga matches through the Mediacoach^®^ multi-camera platform, the only tool in which movement patterns could be collected at that time. The following variables were recorded: total distance (TD) traveled, distance traveled at low-intensity sprint (LIS) (>21 km/h), and high-intensity sprint (HIS) (>24 km/h) in meters [12]

### 2.3. Statistical Analysis

For the descriptive analysis, the absolute frequency (N) and the relative frequency (%) have been calculated for the qualitative variables. In the case of quantitative variables, the mean and standard deviation have been obtained.

For the quantitative variables, the Shapiro–Wilk test of normality was performed. If the behavior of the variables follows a normal distribution, the Mann–Whitney U-test for independent samples was applied to describe the differences in physical demand variables and injury incidence, depending on the studied season. For the player’s position, a Kruskal–Wallis test for independent samples was applied.

Finally, a correlation analysis was also carried out to assess the intensity and trend in the relationship between the quantitative variables. Depending on the behavior of the variables, either the Pearson correlation coefficient or the Spearman correlation coefficient was used.

A 95% confidence level was considered so that the experimental *p*-value was compared with a significance level of *p* < 0.05. The statistical analysis was performed using the IBM SPSS Statistics for Windows, version 22 (IBM Corp., Armonk, NY, USA).

## 3. Results

The mean age of the players was 26 ± 3.8 years presenting a Body Mass Index (BMI) of 23.8 ± 1.1 Kg/m^2^ and a mean of 5.0 ± 3.6 years as professional soccer players.

The players covered a TD during the two seasons’ matches that averaged to 9429.1 ± 1462.8 m. In this period, distance traveled at HIS was 201.2 ± 112.2 m and distance traveled at LIS 221.3 ± 80.2 m. Performance and injury data showed asymmetric distribution and therefore nonparametric tests were applied for analyzing differences between seasons and per player position.

### 3.1. Physical Demand Variables and Injury Incidence Analysed between Seasons and by Player Position

There are statistically significant differences between the two seasons in the TD (*p* = 0.006), the distance traveled in the second season and the distance traveled at a HIS (*p* = 0.007), being greater in the second season. Table 1 provides data about the physical demands variables and injury incidence and the differences between the two seasons.

The TD was different according to the playing position; the differences being statistically significant between the various positions (Table 2). Fullbacks (FB) and central-midfielders (CM) covered more meters in TD (9962.7 ± 883.7 and 10,084.0 ± 1509.3 respectively) than the rest of the players in other positions, the difference being statistically significant with central backs (CB) and wide-midfielders (WM) of *p* < 0.001. The CB (8772.8 ± 1080.0) and the WM (8220.7 ± 1681.9) covered fewer meters than the rest of the players from other positions (*p* < 0.001).

For the distance covered in a HIS, the same results were obtained, except between the WMs and Forwards (Fs), where no significant differences were detected (*p* = 0.135).

For the LIS distance, there are also statistically significant differences between positions, except between central-midfielders and Fs (*p* = 0.370). With regard to the HIS, the WMs covered more meters (316.4 ± 115.3) and the CMs less (121.6 ± 57.5; *p* < 0.001). In LIS, the Fs traveled more meters (281.5 ± 79.0), while the CBs traveled the least (170.3 ± 54.3; *p* < 0.001).

Table 2 includes data about injuries between the two studied seasons. During both seasons, there was an average of 4.7 ± 2.2 injuries, being higher in training (2.9 ± 1.6) than in matches (1.7 ± 1.7). As shown, there are no statistically significant differences in the number of injuries, the severity or the type of injury between the two seasons, except for severe injuries (>28 days) which is higher in the second studied season (*p* = 0.01).

### 3.2. Differences between Seasons in Injury Incidence and Physical Demand Variables Analyzed by Player Position

The CB showed statistically significant differences in the incidence between season one (S1) (19.1 ± 5.1) and season two (S2) (12.7 ± 5.4) (*p* = 0.048). For the FB position, there were statistically significant differences in the HIS between S1 and S2, being 214.0 ± 93.9 m and 306.5 ± 124.1 m, respectively (*p* < 0.001); for LIS (S1 = 257.8 ± 61.5 and S2 = 305.2 ± 87.5) (*p* < 0.001) as well as the incidence of injury during training (S1 = 6.8 ± 3.8 and S2 = 3.6 ± 2.5) (*p* = 0.048), with the joint-ligamentary injuries incidence being different between seasons (S1 = 1.5 ± 1.0 and S2 = 0.3 ± 0.5) (*p* = 0.028). No statistically significant differences were found for the CM between the studied seasons.

No statistically significant differences were found for the CM between the studied seasons.

For the WM, statistically significant differences were found in HIS, being 278.3 ± 110.8 for S1 and 355.7 ± 107.7 for S2 (*p* = 0.001). For the Fs, there were statistically significant differences in TD (S1 = 9685.9 ± 998.4 and S2 = 8949.1 ± 1427.7) (*p* = 0.007).

### 3.3. Correlations between Physical Demand and Injury Incidence by Player Position

High and statistically significant positive correlations were found in the TD for CM in both moderate injuries (r = 0.810; *p* = 0.027) and joint and ligament type (r = 0.802; *p* = 0.030) as well as in the FB and severe injuries (r = 0.791; *p* = 0.034). In meters to HIS, correlations were established for FB and minor injuries (r = 0.849; *p* = 0.016), as well as in CB for the number of total injuries (r = 0.894; *p* = 0.041), moderate injuries (r = 0.894; *p* = 0.041), and those of articular-ligamentary type (r = 0.894; *p* = 0.041). No significant positive correlations were observed for meters at LIS, WM and Fs.

Subsequently, negative correlations were found in the TD for CBs in minor injuries (r = −0.975; *p* = 0.005) and muscle-tendon unit injuries (r = −0.949; *p* = 0.014) and in CMs regarding the number of injuries in training (r = −0.810; *p* = 0.027). In the meters to LIS, only correlations were shown for the FB in the number of injuries in training (r = −0.805; *p* = 0.029), incidence to training (r = −0.800; *p* = 0.031), and total incidence (r = −0.775; *p* = 0.041). No significant negative correlations were observed for meters to HIS, WM, and F.

## 4. Discussion

The present study aimed to describe the injury incidence in both training and matches with physical demand variables, TD and meters to HIS and LIS, according to the field position of professional soccer players during two seasons using a video analysis system. Our results showed statistically significant correlations in CB, FB, and CM for TD, LIS and HIS variables but not for WM or F players. Consequently, the current findings suggest that match-performance data may be useful for detecting injury risks at certain playing positions.

The physical demand data was obtained using the Mediacoach^®^ video-tracking system which, at that time, was the only available tool that could be used in official competition. It is more comfortable for the player, and the data can be compared with that of other teams in the same competition [14,15]. The literature has demonstrated the validity of this system and others such as Amisco or Prozone^®^ [16], although the use of GPS is currently more standardised owing to its versatility and accessibility in daily practice [17].

Some authors have demonstrated the importance of the tactical role of the player within a football team and its relationship to performance [19]. The study of physical variables, such as TD, LIS, and HIS, has also been associated with physical performance and injury incidence [21]. However, some authors have highlighted the importance of other performance variables like acceleration, deceleration, total load, or the calculation of acute:chronic load ratios [26].

The players who covered the longest TD were the CMs. Previous studies have reported this finding in the Spanish league using the Mediacoach^®^ multi-camera video system [13,14]. In our study, a positive correlation was observed with moderate injury severity (8–28 days) and joint injuries. Some authors pointed to the most tactically demanding for CM position. CM has been shown to play an important role in decision-making, triggering mental fatigue which can contribute to an increase in the risk of injury [37,38]. Moreover, CMs run longer TDs to help the team in both defense and attack actions [18]. This demand on CMs during matches is reflected in our results, where a negative correlation between TD and the number of injuries was revealed. In contrast, Dupont stated that in teams that played more than one game per week, both TDs and high-intensity efforts were not significantly affected, but the injury rate was significantly higher [8].

The CBs did not run as many meters in TD or high-intensity stocks as in other positions, suggesting that they are not exposed to covering these physical demands. In the present study, there was a positive correlation in CBs between meters run in HIS and the number of total, moderate severity, and joint-type injuries. On the other hand, there was a negative correlation between TD and the injuries of the muscle-tendon type and minor severity (1–3 days). Therefore, our results coincide with those published by Bush et al. (2016), who showed that in modern football, the CBs could not cover demands from other positions, such as FB [9]. Therefore, this change in position was defined as a risk factor for injury because there are shorter recovery periods between high-intensity efforts in the FB position [12].

In the case of the FBs, the greater the distance they traveled to LIS during the matches, the lower the total incidence and the number of injuries in training. Previous studies related the importance and evolution of high-intensity actions, including very high intensity running (VHSR) and sprint meters with the position of FB [9,21,23]. Other studies also stated that due to the evolution of high-intensity actions, FBs should be well exposed to these position-specific demands as long sprints in lathes at 30 m to improve performance [21]. Therefore, if they are more exposed, they would be more adapted and the risk of injuries would be reduced [29]. Including these actions into training and prevention programs helps professional players to be optimally prepared for such demands imposed by a competitive match play [26,42]. An illustrative example is provided in the case of the players who suffered previous hamstring injuries that corresponded with less covered distance in sprint or high-velocity actions [4,28]. With regard to the HIS meters, our results showed that the FBs traveled longer distance and had minor injuries (severity, 1–3 days) that do not lead to absence from matches.

With regard to TD, a positive relationship with severe injuries was observed in this group of players, although current research has downplayed the importance of this parameter about high-intensity actions, which has been considered a better indicator of performance and therefore of risk of injury [23].

No statistically significant correlations were found for the WM and F positions regarding physical demands and injury incidence. Specifically, our WM players, as in other La Liga studies, have shown the highest values in meters to HIS and have been behind the FBs who have performed the most meters to LIS [21]. Therefore, we believe that a lower incidence of injury could be justified for the greater exposure to this type of effort during training and matches.

Based on the data obtained through video analysis systems, controlled training sessions can be programmed to expose players to demands that are comparable to those of a match, thus trying to reduce the risk of injury [43]. As Impellizzeri et al. [44] stated, athletic performance and injury prevention are dependent, and, for this reason, the communication between medical and technical staff should be highly coordinated to improve performance while trying to minimise the risk of injury.

Differences in participation in official games, as in our second studied season, can cause significant imbalances in the chronic external loads between players in a team [45]. This situation should be considered and minimised in training sessions to prevent imbalances in workload for those who usually play or not. Technical staff involved in planning training for performance and injury prevention usually monitor the acute:chronic workload ratio, and the data of MediaCoach^®^ contribute to its calculation. From this ratio analysis, preventive interventions should focus on increasing chronic exposure to load and avoid spikes that exceed 2.0 [46].

The findings of this study should be interpreted with caution due to certain methodological limitations. First, while video tracking systems have been the most widely used systems in the analysis of players’ physical demands for some time, the recent incorporation of GPS technology has gained ground. GPS technology consists of more accurate devices and can be used to control the load in training and matches, especially the relevant data about accelerations and decelerations.

Second, this is an observational study with an exploratory aim, and causality or any form of relationship cannot be established. In addition, the small sample size reduces the reliability of the results. It would be interesting in the future to extend the analysis with Machine Learning analysis techniques to predict and establish relationships between performance improvement, injury programs, and injury incidence.

Third, due to the use of a video analysis system, the workload performed by the soccer players during training was not registered and considered in the study, which could influence the performance values in matches.

## 5. Conclusions

This study shows differences in injury incidence and physical demand variables collected with the Mediacoach^®^ system in the positions of CB, FB, and CM, but not for WM and F.

Specifically, relationships were established between TD and WM for moderate injuries of the articular-ligament type and in the number of injuries in training and in minor and muscle-tendinous injuries for CB. For the LIS, relations were observed for the FB in the number of injuries during training, in the total incidence of minor severity injuries and in injuries located in the foot and the lumbopelvic region.

Finally, in the meters run in HIS, FB was correlated to minor severity injuries and in CB, with the number of total injuries, moderate severity injuries and those injuries of the articular-ligamentary type. The data provided could help medical services and technical staff in preventing injuries by planning specific actions by player position.

## Figures and Tables

**Table 1:** Differences in the physical demands variables and injury incidence between two seasons.

Variables		SEASON 2012/2013 (S1)	SEASON 2013/2014 (S2)	GLOBAL	*p*-Value
TD (meters)		9579.9 ± 1436.7	9274.4 ± 1475.4	9429.1 ± 1.462.8	0.006 *
HIS (meters)		186.9 ± 100.6	215.9 ± 121.4	201.2 ± 112.2	0.007 *
LIS (meters)		219.2 ± 74.0	223.5 ± 86.1	221.3 ± 80.2	0.089
Mean number of injuries		5.0 ± 2.5	4.6 ± 2.1	4.7 ± 2.2	0.583
Exposure of injuries	Training	3.4 ± 2.0	2.7 ± 1.3	2.9 ± 1.6	0.233
	Matches	1.4 ± 1.5	1.9 ± 1.8	1.7 ± 1.7	0.231
Severity of injuries	<3 days	2.3 ± 1.7	2.0 ± 1.5	2.1 ± 0.5	0.504
	4–7 days	1.0 ± 1.2	0.9 ± 0.8	0.9 ± 0.9	0.856
	8–28 days	1.4 ± 1.1	1.1 ± 1.1	1.2 ± 1.1	0.317
	>28 days	0.3 ± 0.5	0.7 ± 0.6	0.6 ± 0.6	0.010 *
Type of injuries	Muscle-tendinous	2.8 ± 2.1	2.4 ± 1.4	2.5 ± 1.6	0.691
	Joint-ligamentary	1.6 ± 1.0	1.3 ± 1.3	1.4 ± 1.2	0.235
	Bone	0.4 ± 0.5	0.7 ± 1.4	0.6 ± 1.2	0.842
	Other type	0.3 ± 0.7	0.2 ± 0.4	0.2 ± 0.5	0.759
Total incidence (injuries per 1000 h of exposure)	Training	10.3 ± 6.2	7.8 ± 3.8	8.5 ± 4.7	0.029 *
	Match	15.3 ± 16.5	22.7 ± 21.3	20.5 ± 20.2	0.071
	Total incidence	11.9 ± 6.0	10.5 ± 4.9	10.9 ± 5.2	0.180

Data are presented as a mean ± standard deviation; Abbreviations: TD: total distance traveled; HIS; distance traveled at a high-intensity sprint; LIS: distance traveled at a low-intensity sprint. Mann–Whitney U test for independent samples; * (*p* < 0.05).

**Table 2:** Differences in the physical demands variables and injury incidence between the player’s position.

Variables		Central Back	Fullback	Central- Midfielder	Wide- Midfielder	Forward	Global
TD (meters)		8772.8 ± 1080.0	9962.7 ± 883.7	10,084.0 ± 1509.3	8220.7 ± 1681.9	9317.5 ± 1280.5	9429.1 ± 1462.8 **
HIS (meters)		155.5 ± 64.4	260.3 ± 119.1	121.6 ± 57.5	316.4 ± 115.3	230.3 ± 92.1	201.2 ± 112.2 **
LIS (meters)		170.3 ± 54.3	281.5 ± 79.0	209.1 ± 73.1	241.0 ± 73.6	214.9 ± 73.7	221.3 ± 80.2 **
Mean number of injuries		6.5 ± 2.5	2.8 ± 1.3	4.8 ± 1.9	5.3 ± 2.3	4.1 ± 1.5	4.7 ± 2.2 *
Exposure of injuries	Training	3.6 ± 1.3	1.6 ± 1.1	3.0 ± 1.2	3.9 ± 2.5	2.8 ± 1.3	2.9 ± 1.6 *
	Matches	2.8 ± 2.0	1.3 ± 1.2	1.7 ± 2.0	1.4 ± 1.3	1.5 ± 1.1	1.7 ± 1.7
Severity of injuries	<3 days	2.7 ± 1.4	0.8 ± 1.0	2.2 ± 1.7	3.3 ± 0.9	1.6 ± 1.2	2.1 ± 1.5 **
	4–7 days	1.3 ± 1.1	0.7 ± 1.2	0.9 ± 0.7	0.8 ± 0.7	1.0 ± 0.9	0.9 ± 0.9
	8–28 days	2.2 ± 1.3	1.0 ± 1.0	1.2 ± 0.7	0.9 ± 1.4	0.8 ± 0.8	1.2 ± 1.1 *
	>28 days	0.6 ± 0.5	0.4 ± 0.5	0.7 ± 0.7	0.4 ± 0.5	0.7 ± 0.5	0.6 ± 0.6
Type of injuries	Muscle-tendinous	3.9 ± 2.2	1.6 ± 1.1	2.3 ± 1.1	2.5 ± 1.2	2.3 ± 1.9	2.5 ± 1.6 **
	Joint-ligamentary	1.5 ± 1.0	0.7 ± 0.9	1.6 ± 1.1	2.3 ± 1.9	1.2 ± 1.0	1.4 ± 1.2
	Bone	1.1 ± 1.0	0.5 ± 0.5	0.7 ± 1.8	0.0 ± 0.0	0.6 ± 0.8	0.6 ± 1.2 *
	Other	0.3 ± 0.7	0.2 ± 0.4	0.3 ± 0.6	0.4 ± 0.5	0.0 ± 0.0	0.2 ± 0.5
Total incidence (injuries/1000 h of exposure)	Training	10.3 ± 4.3	4.7 ± 3.3	8.8 ± 6.5	11.4 ± 7.6	8.1 ± 3.8	8.5 ± 4.7
	Match	31.4 ± 22.7	14.9 ± 14.0	20.0 ± 24.8	16.8 ± 16.1	18.1 ± 13.3	20.5 ± 20.2
	Total	14.9 ± 6.0	6.7 ± 3.0	11.1 ± 4.5	12.3 ± 5.5	9.6 ± 3.5	10.9 ± 5.2

Data are presented as a mean ± standard deviation. Abbreviations: TD, total traveled distance; HIS, distance traveled running at a high-intensity sprint; LIS, distance traveled running at a low-intensity sprint. Kruskal–Wallis test for independent samples * (*p* < 0.05); ** (*p* < 0.001).

## Data Availability

The authors confirm that the datasets analyzed during the study are available from the first au-thor or the corresponding author upon reasonable request.
